# Efficacy and safety of intravesical instillation of KRP‐116D (50% dimethyl sulfoxide solution) for interstitial cystitis/bladder pain syndrome in Japanese patients: A multicenter, randomized, double‐blind, placebo‐controlled, clinical study

**DOI:** 10.1111/iju.14505

**Published:** 2021-02-12

**Authors:** Naoki Yoshimura, Yukio Homma, Hikaru Tomoe, Atsushi Otsuka, Takeya Kitta, Naoya Masumori, Yoshiyuki Akiyama, Aya Niimi, Takahiko Mitsui, Masaharu Nanri, Takashige Namima, Mineo Takei, Akito Yamaguchi, Yuki Sekiguchi, Mitsuru Kajiwara, Shinya Kobayashi, Kaname Ameda, Yozo Ohashi, Sadaaki Sakamoto, Osamu Muraki, Toshihide Shishido, Shinji Kageyama, Koji Kokura, Homare Okazoe, Tomonori Yamanishi, Toyohiko Watanabe, Takashi Uno, Akira Ohinata, Tomohiro Ueda

**Affiliations:** ^1^ Department of Urology University of Pittsburgh School of Medicine Pittsburgh PA USA; ^2^ Department of Urology Ueda Clinic Kyoto Japan; ^3^ Japanese Red Cross Medical Center Tokyo Japan; ^4^ Department of Pelvic Reconstructive Surgery/Urology Tokyo Women’s Medical University Medical Center East Tokyo Japan; ^5^ Department of Urology Hamamatsu University School of Medicine Shizuoka Japan; ^6^ Department of Renal and Genitourinary Surgery Graduate School of Medical Science Hokkaido University Hokkaido Japan; ^7^ Department of Urology Sapporo Medical University School of Medicine Hokkaido Japan; ^8^ Department of Urology Graduate School of Medicine The University of Tokyo Tokyo Japan; ^9^ National Center for Global Health and Medicine Tokyo Japan; ^10^ Department of Urology University of Yamanashi Graduate School of Medical Sciences Yamanashi Japan; ^11^ Nanri Urological Clinic Saga Japan; ^12^ Department of Urology Tohoku Rosai Hospital Miyagi Japan; ^13^ Department of Urology Harasanshin Hospital Fukuoka Japan; ^14^ Female Urology Women’s Clinic LUNA Next Stage Kanagawa Japan; ^15^ Department of Urology Hiroshima Prefectural Hospital Hiroshima Japan; ^16^ Miyanosawa Nephrourological Clinic Hokkaido Japan; ^17^ Hokkaido Memorial Hospital of Urology Hokkaido Japan; ^18^ Department of Urology Japan Community Healthcare Organization Ritsurin Hospital Kagawa Japan; ^19^ Department of Urology Nakamura Hospital Oita Japan; ^20^ Department of Urology Fujita General Hospital Fukushima Japan; ^21^ Department of Urology Tokyo Medical University Hachioji Medical Center Tokyo Japan; ^22^ Kageyama Urology Clinic Shizuoka Japan; ^23^ Department of Urology Takarazuka City Hospital Hyogo Japan; ^24^ Kokura Urology Clinic Hyogo Japan; ^25^ Department of Urology KKR Takamatsu Hospital Kagawa Japan; ^26^ Department of Urology Continence Center Dokkyo Medical University Tochigi Japan; ^27^ Department of Urology Okayama University Graduate School of Medicine, Dentistry and Pharmaceutical Sciences Okayama Japan; ^28^ Clinical Development Center Kyorin Pharmaceutical Co., Ltd. Tokyo Japan

**Keywords:** bladder‐centric phenotype, bladder pain syndrome, dimethyl sulfoxide, interstitial cystitis, randomized controlled trial

## Abstract

**Objective:**

To evaluate the efficacy and safety of intravesical KRP‐116D, 50% dimethyl sulfoxide solution compared with placebo, in interstitial cystitis/bladder pain syndrome patients.

**Methods:**

Japanese interstitial cystitis/bladder pain syndrome patients with an O’Leary‐Sant Interstitial Cystitis Symptom Index score of ≥9, who exhibited the bladder‐centric phenotype of interstitial cystitis/bladder pain syndrome diagnosed by cystoscopy and bladder‐derived pain, were enrolled. Patients were allocated to receive either KRP‐116D (*n* = 49) or placebo (*n* = 47). The study drug was intravesically administered every 2 weeks for 12 weeks.

**Results:**

For the primary endpoint, the change in the mean O’Leary‐Sant Interstitial Cystitis Symptom Index score from baseline to week 12 was −5.2 in the KRP‐116D group and −3.4 in the placebo group. The estimated difference between the KRP‐116D and placebo groups was −1.8 (95% confidence interval −3.3, −0.3; *P* = 0.0188). Statistically significant improvements for KRP‐116D were also observed in the secondary endpoints including O’Leary‐Sant Interstitial Cystitis Problem Index score, micturition episodes/24 h, voided volume/micturition, maximum voided volume/micturition, numerical rating scale score for bladder pain, and global response assessment score. The adverse drug reactions were mild to moderate, and manageable.

**Conclusions:**

This first randomized, double‐blind, placebo‐controlled trial shows that KRP‐116D improves symptoms, voiding parameters, and global response assessment, compared with placebo, and has a well‐tolerated safety profile in interstitial cystitis/bladder pain syndrome patients with the bladder‐centric phenotype.

**Abbreviations & Acronyms**
ADR: adverse drug reaction
AE: adverse event
CI: confidence interval
BPS: bladder pain syndrome
DMSO: dimethyl sulfoxide
GRA: global response assessment
IC: interstitial cystitis
ICPI: O’Leary‐Sant Interstitial Cystitis Problem Index
ICSI: O’Leary‐Sant Interstitial Cystitis Symptom Index
LS: least squares
NRS: numerical rating scale
PRO: patient‐reported outcome
SD: standard deviation
TEAE: treatment‐emergent adverse event

## Introduction

IC/BPS is a chronic bladder condition characterized by bladder pain, frequency, and urgency in the absence of other well‐defined pathologies.^1^, ^2^, ^3^ The etiology is not well understood, nor is there a widely accepted definition.^4^ Pharmacotherapy is very limited, and the drugs approved by the U.S. Food and Drug Administration for IC/BPS are confined to pentosan polysulfate sodium and a 50% w/w aqueous solution of DMSO solution. In Japan, no drugs indicated for IC/BPS have been approved. Despite intensive studies over the past decades, development of novel drugs has largely failed, partly due to confusion over combining phenotypes under one umbrella,^5^, ^6^, ^7^, ^8^ as well as lack of efficacy. Also, Hunner lesion IC/BPS, the incidence of which is reportedly up to 57% among IC/BPS patients, has again begun to attract attention, and is categorized as a separate disease from non‐Hunner IC/BPS.^9^, ^10^ Therefore, the classification of either a bladder‐centric phenotype of IC/BPS identified by Hunner lesions or hydrodistention‐induced glomerulations, or an extra‐bladder phenotype of IC/BPS that overlaps with other chronic pain conditions could lead to the development of phenotype‐specific treatment methods for IC/BPS.^11^

Rimso‐50^®^ (50% DMSO solution) was approved in 1978 in the USA, and to date, the American Urological Association Guideline for IC/BPS recommends the intravesical instillation of DMSO as a second‐line treatment.^2^ However, the European Association of Urology guidelines on chronic pelvic pain do not recommend its use because of insufficient evidence.^12^ Although many clinical trials have demonstrated the clinical benefits of 50% DMSO in the treatment of IC/BPS,^13^, ^14^ no well‐controlled randomized placebo‐controlled double‐blind parallel comparison studies have been reported.

The aim of the present study was to evaluate the efficacy and safety of intravesical instillation of KRP‐116D (50% DMSO solution) in IC/BPS patients with the bladder‐centric phenotype identified by cystoscopic examination and bladder pain relief after intravesical lidocaine instillation.

The study was registered at Japic‐Clinical Trials Information (JapicCTI‐173566).

## Methods

### Study design and participants

This was a multicenter, randomized, double‐blind, placebo‐controlled parallel‐group comparative trial. The study protocol and informed consent form were approved by the institutional review board at each participating study site. All patients gave written informed consent before initiation of any study‐specific procedures. The study was conducted in accordance with the ethical principles originating in or derived from the Declaration of Helsinki and Good Clinical Practice Guidelines. The study was designed and conducted by the sponsor (Kyorin Pharmaceutical Co., Ltd., Tokyo, Japan) in collaboration with the principal investigators. The sponsor monitored the study conduct, collected the data, and performed the statistical analyses.

The study was conducted at 24 sites in Japan from May 2017 to July 2018. This study consisted of a 1‐ to 4‐week screening period, a 2‐week single‐blind placebo run‐in period and a 12‐week double‐blind treatment period (Fig. S1). Japanese IC/BPS patients aged ≥20 years with an ICSI score of ≥9, who exhibited the bladder‐centric phenotype of IC/BPS, were enrolled in this study. Patients who met the eligibility criteria (Table S1) entered the 2‐week placebo run‐in period. Once eligibility was confirmed before randomization, patients were randomized 1:1 to receive either KRP‐116D or placebo by the minimization method, which was adjusted centrally by dynamic assignment, with ICSI score (9 ≤ to <13, 13 ≤ to <17, or 17 ≤ to ≤20) assessed at week 0 as the assignment factor. The randomization sequence was generated independently of the study sponsor. Treatment allocation of patients was initiated via website. The investigators, patients and sponsor were masked to the treatment assignment. To ensure that masking was maintained, KRP‐116D and placebo vials for intravesical instillation were manufactured as identical in their appearance. Patients received 50 mL of the assigned study drug intravesically every 2 weeks for 12 weeks. During the run‐in and treatment periods, before administration of the study drug, residual urine in the bladder was removed using a urethral catheter, and lidocaine solution (4%, 20 mL) was instilled and left in the bladder for 5–15 min, and then withdrawn through the catheter. Subsequently, 50 mL of the study drug was intravesically administered and left in the bladder for at least 15 min according to the package insert of Rimso‐50® and, thereafter, the patients were instructed to spontaneously urinate to expel the solution inside the bladder.

### Efficacy and safety assessments

For efficacy assessment, patients were asked to complete the ICSI and the ICPI questionnaires. Voided volume and number of micturitions/24 h were assessed using a 2‐day patient voiding diary, and NRS for bladder pain using a 3‐day diary. Patients also completed a GRA questionnaire at week 12, which is a seven‐point symmetric scale: markedly improved, moderately improved, slightly improved, no change, slightly worse, moderately worse, and markedly worse. Patients whose condition was moderately improved or markedly improved were defined as responders. The primary endpoint was change in ICSI score at week 12 from baseline. The secondary endpoints were changes at week 12 from baseline in ICPI score, mean number of micturitions/24 h, mean voided volume/micturition, maximum voided volume/micturition, and mean NRS score for bladder pain, and GRA score at week 12. The other endpoints were changes in these variables from baseline to each visit. Safety was assessed based on AEs, vital signs, 12‐lead electrocardiogram, clinical tests, and ophthalmic examination.

### Statistical analysis

According to the reports, the mean change in ICSI from baseline to week 12 was estimated to be −4.5 in the KRP‐116D group and −2.5 in the placebo group, with an SD of 3.0 in both groups.^15^, ^16^ A sample size of 37 patients/group provided 80% power to demonstrate the superiority of KRP‐116D over placebo with a two‐sided significance level of 5% on comparison using Student’s *t*‐test. Therefore, in this study, the plan was to enroll 45 patients/group (total of 90 patients), taking the possibility of dropouts into account.

The safety analysis was performed for patients in the safety analysis set. The primary set for the efficacy assessment was the full analysis set and secondarily the per‐protocol set (Table S2). Summary statistics of the change in the efficacy variables at week 12 from baseline were calculated in each group. The LS mean, difference in the LS mean, and two‐sided 95% CI of changes in the efficacy variables from baseline to time of assessment in each group were estimated using a mixed model for repeated measures The difference in the GRA responder rate between groups was calculated, and the difference between groups was determined using Fisher’s exact test.

AEs that occurred after administration of the study drug were analyzed as TEAEs. ADRs were defined as TEAEs related to the study drug. Ophthalmic examinations included a corrective vision test, a refraction test, and slit lamp microscopy. AEs were coded using the Medical Dictionary for Regulatory Activities, Japanese version, 20.0.

All statistical tests used a significance level of 5% and were two‐sided. All analyses were performed using SAS software version 9.4 for Windows (SAS Institute, Cary, NC, USA).

## Results

### Patients

**Fig. 1:**
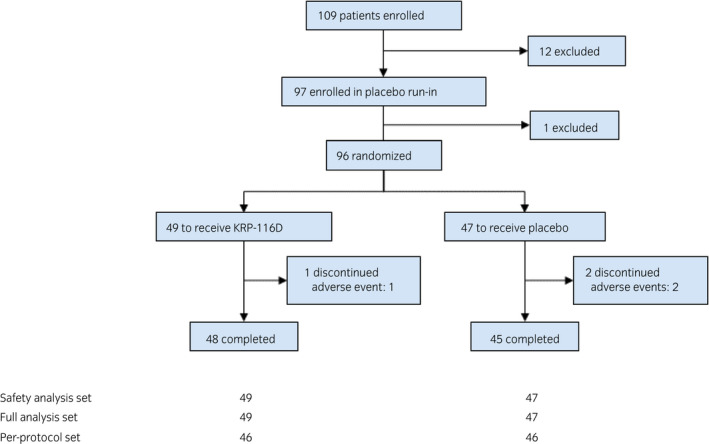
Patient disposition.

The 109 IC/BPS patients who gave informed consent were confirmed as having the bladder‐centric IC/BPS phenotype with Hunner lesions or other pathologies, such as hydrodistention‐induced glomerulations, according to the screening criteria including cystoscopy. Among them, 97 patients met the inclusion criteria at Visit 2 (Table S1). Also, before randomization, all study participants exhibited short‐term relief from bladder pain after intravesical lidocaine instillation during the placebo run‐in period, confirming that their symptoms originated in the bladder. A total of 96 patients were randomized to receive either KRP‐116D (*n* = 49) or placebo (*n* = 47; Fig. 1). Baseline demographics and disease characteristics were well balanced between the two groups (Table 1).

**Table 1:** Summary of demographic and other baseline characteristics

	KRP‐116D (*n* = 49)	Placebo (*n* = 47)
Sex, *n* (%)		
Male	6 (12.2)	3 (6.4)
Female	43 (87.8)	44 (93.6)
Age		
<65 years, *n* (%)	21 (42.9)	19 (40.4)
≥65 years, *n* (%)	28 (57.1)	28 (59.6)
Mean ± SD	63.6 ± 14.2	64.5 ± 13.5
(Minimum, median, maximum)	(30, 66.0, 87)	(36, 67.0, 87)
Height, cm		
Mean ± SD	155.14 ± 8.58	154.28 ± 7.50
(Minimum, median, maximum)	(139.0, 152.90, 179.7)	(135.4, 154.10, 171.1)
Weight		
<50 kg, *n* (%)	20 (40.8)	19 (40.4)
≥50 kg, *n* (%)	29 (59.2)	28 (59.6)
Mean ± SD	55.80 ± 11.79	52.88 ± 11.52
(Minimum, median, maximum)	(38.6, 53.60, 90.0)	(29.6, 52.80, 86.8)
Body mass index		
<25.0 kg/m^2^, *n* (%)	37 (75.5)	39 (83.0)
≥25.0 kg/m^2^, *n* (%)	12 (24.5)	8 (17.0)
Mean ± SD	23.08 ± 3.87	22.12 ± 4.18
(Minimum, median, maximum)	(17.6, 22.83, 34.9)	(14.5, 21.39, 36.0)
Type of IC, *n* (%)		
Hunner type	42 (85.7)	41 (87.2)
Non‐Hunner type	7 (14.3)	6 (12.8)
ICSI		
Mean ± SD	13.9 ± 3.1	13.7 ± 3.1
(Minimum, median, maximum)	(9, 14.0, 20)	(9, 13.0, 20)
ICPI		
Mean ± SD	12.0 ± 3.2	11.8 ± 3.0
(Minimum, median, maximum)	(4, 13.0, 16)	(6, 12.0, 16)
Mean number of micturitions per 24 h†		
Mean ± SD	16.41 ± 9.41	14.93 ± 5.72
(Minimum, median, maximum)	(8.0, 15.00, 56.5)	(8.0, 13.50, 37.0)
Mean voided volume per micturition (mL)†		
Mean ± SD	109.4 ± 54.8	114.1 ± 58.5
(Minimum, median, maximum)	(27, 105.8, 257)	(31, 104.4, 305)
Maximum voided volume per micturition (mL)†		
Mean ± SD	183.5 ± 104.2	184.4 ± 104.0
(Minimum, median, maximum)	(50, 185.0, 600)	(45, 165.0, 500)
NRS for bladder pain^‡^		
Mean ± SD	6.50 ± 1.46	6.51 ± 1.50
(Minimum, median, maximum)	(4.0, 6.33, 10.0)	(4.0, 6.33, 10.0)
History of hydrodistention, *n* (%)		
No	6 (12.2)	9 (19.1)
Yes	43 (87.8)	38 (80.9)
<1 year	18 (36.7)	15 (31.9)
≥1 year	25 (51.0)	23 (48.9)
History of DMSO intravesical instillation, *n* (%)		
No	43 (87.8)	42 (89.4)
Yes	6 (12.2)	5 (10.6)
Use of drug for primary disease at screening, *n* (%)		
No	9 (18.4)	16 (34.0)
Yes	40 (81.6)	31 (66.0)
Antihistamine	4 (8.2)	3 (6.4)
Antidepressants/antipsychotics	8 (16.3)	12 (25.5)
Suplatast tosylate	14 (28.6)	17 (36.2)
Systemic steroids	2 (4.1)	0 (0.0)
Acidic urine remedy	3 (6.1)	4 (8.5)
Drugs for overactive bladder/urination disorder	9 (18.4)	12 (25.5)
Analgesics	23 (46.9)	16 (34.0)
Others	10 (20.4)	10 (21.3)

Analysis set: full analysis set.

† Mean of the latest 2 days before week 0.

‡ Mean of the latest 3 days before week 0.

### Efficacies

**Fig. 2:**
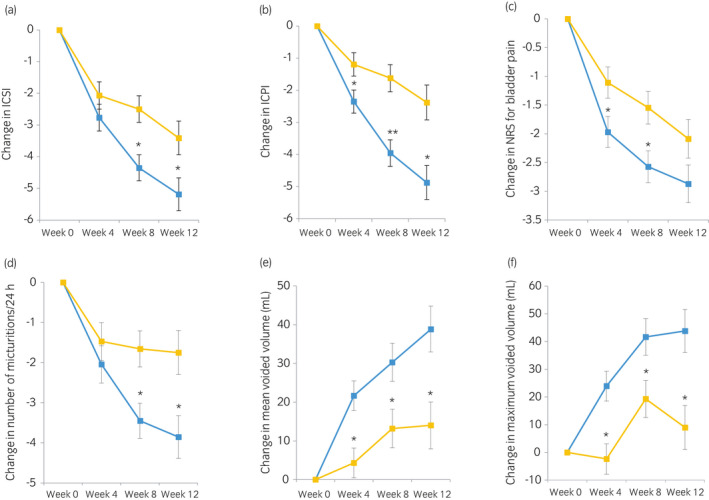
LS mean change (standard error) in IC/BPS symptoms and voiding parameters from baseline at each visit in the KRP‐116D and placebo groups with respect to: (a) ICSI; (b) ICPI; (c) NRS for bladder pain; (d) number of micturitions/24 h; (e) voided volume/micturition (mL); and (f) maximum voided volume/micturition (mL). The differences in the primary and secondary efficacy variables for the KRP‐116D and placebo groups were calculated using a mixed model for repeated measures. *P < 0.05, **P < 0.001. 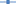 KRP‐116D and 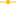 placebo.

For the primary endpoint, the change in the LS mean ICSI score from baseline to week 12 was −5.2 in the KRP‐116D group and −3.4 in the placebo group (Table 2, Fig. 2). The estimated difference between the KRP‐116D and placebo groups was −1.8 (*P* = 0.0188), indicating the superiority of KRP‐116D over placebo (Table 2). Similar results were obtained in the per‐protocol set population. With respect to the secondary endpoints, compared to placebo, significant improvements in ICPI and voiding parameters, including micturitions/24 h, voided volume/micturition, and maximum voided volume/micturition, were seen in patients treated with KRP‐116D (Fig. 2, Table 3, Table S2). No statistically significant difference in pain score change between DMSO and placebo was observed at week 12; however, significant differences were found at weeks 4 and 8. The GRA response rates were higher in the KRP‐116D group than in the placebo group (Table 4).

**Table 2:** Change in ICSI score from baseline to week 12

	ICSI total score				Change in ICSI total score from baseline to week 12			
	Week 0		Week 12					
	*n*	Mean ± SD	*n*	Mean ± SD	Median	Mean ± SD	LS mean	95% CI
KRP‐116D	49	13.9 ± 3.1	48	8.7 ± 4.2	−5.0	−5.2 ± 4.4	−5.2	(−6.2, −4.2)
Placebo	47	13.7 ± 3.1	45	10.3 ± 4.0	−4.0	−3.4 ± 2.9	−3.4	(−4.5, −2.4)

Estimated difference between the groups	Difference in LS means	95% CI for difference in LS means	*P*
KRP‐116D *vs* placebo	−1.8	(−3.3, −0.3)	0.0188

Analysis set: full analysis set.

**Table 3:** Changes in efficacy variables from baseline to week 12

	KRP‐116D	Placebo
	(*n* = 49)	(*n* = 47)
ICPI		
No. of subjects at week 12	48	45
LS mean change at week 12 from baseline	−4.9	−2.4
95% CI	(−5.9, −3.8)	(−3.5, −1.3)
Estimated difference	−2.5	–
95% CI for estimated difference	(−4.0, −1.0)	–
*P*‐value	0.0014	–
Number of micturitions per 24 h		
No. of subjects at week 12	48	45
LS mean change at week 12 from baseline	−3.86	−1.75
95% CI	(−4.91, −2.80)	(−2.83, −0.67)
Estimated difference	−2.11	–
95% CI for estimated difference	(−3.62, −0.60)	–
*P*‐value	0.0068	–
Voided volume per micturition		
No. of subjects at week 12	48	45
LS mean change at week 12 from baseline, mL	38.8	14.0
95% CI	(27.1, 50.6)	(2.0, 26.0)
Estimated difference	24.8	–
95% CI for estimated difference	(8.0, 41.6)	–
*P*‐value	0.0042	–
Maximum voided volume per micturition		
No. of subjects at week 12	48	45
LS mean change at week 12 from baseline, mL	43.8	9.0
95% CI	(28.4, 59.2)	(−6.8, 24.7)
Estimated difference	34.8	–
95% CI for estimated difference	(12.8, 56.8)	–
*P*‐value	0.0023	–
Change of maximum voided volume per micturition		
No. of subjects at week 12	48	45
LS mean change at week 12 from baseline, %	29.89	11.54
95% CI	(20.09, 39.70)	(1.55, 21.54)
Estimated difference	18.35	–
95% CI for estimated difference	(4.34, 32.35)	–
*P*‐value	0.0108	–
NRS score for bladder pain		
No. of subjects at week 12	48	45
LS mean change at week 12 from baseline	−2.87	−2.09
95% CI	(−3.52, −2.22)	(−2.75, −1.42)
Estimated difference	−0.78	–
95% CI of estimated difference	(−1.71, 0.15)	–
*P*‐value	0.0973	–

Analysis set: full analysis set.

**Table 4:** GRA at week 12 in the KRP‐116D and placebo groups

	KRP‐116D *n* = 49	Placebo *n* = 47
Frequency of GRA (post‐dose), *n* (%)		
1: Markedly improved	12 (25.0)	5 (10.9)
2: Moderately improved	13 (27.1)	9 (19.6)
3: Slightly improved	15 (31.3)	21 (45.7)
4: No change	4 (8.3)	11 (23.9)
5: Slightly worse	2 (4.2)	0 (0.0)
6: Moderately worse	2 (4.2)	0 (0.0)
7: Markedly worse	0 (0.0)	0 (0.0)
Responder (1 + 2)	25 (52.1)	14 (30.4)
Non‐responder (3 + 4 + 5 + 6 +7)	23 (47.9)	32 (69.6)
Difference between the groups		
Difference (%)	21.6	
95% CI	(1.8, 39.2)	
*P*‐value (Fisher's exact test)	0.0385	

Analysis set: full analysis set.

### Safety

The overall incidences of TEAEs in the KRP‐116D and placebo groups were 69.4% (34/49) and 59.6% (28/47), respectively (Table 5). The incidences of ADRs in the KRP‐116D and placebo groups were 59.2% (29/49) and 27.7% (13/47), respectively. The severity of all ADRs was mild to moderate. No deaths were reported. Other serious AEs included femur fracture (*n* = 1) and vertigo (*n* = 1) in the KRP‐116D group, and cerebral infarction (*n* = 1) in the placebo group. None of the serious AEs were considered related to the study drug. No ocular toxicities were reported. The incidence of TEAEs at the time of administration in the KRP‐116D and placebo groups was 61.2% (30/49) and 31.9% (15/47), respectively. The incidence was highest during the first administration period (week 0 to week 2) in both groups, and decreased gradually over the subsequent administrations (Table S3). The median onset time of TEAEs was 1 day after administration in both groups. In the KRP‐116D group, the incidence of abnormal breath and/or skin odour was 6.1% (3/49); breath odour (*n* = 2), skin odour abnormal (*n* = 1), whereas there were no reports of abnormal odour in the placebo group. Breath odour was an objective finding reported by the investigators/study staff, but was not reported by the patients.

**Table 5:** TEAEs and ADRs

	TEAEs				ADRs			
	KRP‐116D (*n* = 49)		Placebo (*n* = 47)		KRP‐116D (*n* = 49)		Placebo (*n* = 47)	
	*n* (%)	Events	*n* (%)	Events	*n* (%)	Events	*n* (%)	Events
Any AEs	34 (69.4)	142	28 (59.6)	61	29 (59.2)	105	13 (27.7)	25
Any serious AEs	2 (4.1)	–	1 (2.1)	–	0 (0.0)	–	0 (0.0)	–
Any AEs leading to drug withdrawal	1 (2.0)	–	2 (4.3)	–	0 (0.0)	–	1 (2.1)	–
Most common AEs (preferred term incidence in ≥3 patients in any group)								
Viral upper respiratory tract infection	6 (12.2)	7	8 (17.0)	9	0 (0.0)	0	0 (0.0)	0
Contusion	3 (6.1)	3	1 (2.1)	1	0 (0.0)	0	0 (0.0)	0
Bladder discomfort	4 (8.2)	6	1 (2.1)	1	4 (8.2)	6	1 (2.1)	1
Bladder irritation	5 (10.2)	8	1 (2.1)	1	5 (10.2)	8	1 (2.1)	1
Bladder pain	15 (30.6)	47	10 (21.3)	15	15 (30.6)	47	9 (19.1)	13
Pollakiuria	4 (8.2)	9	1 (2.1)	1	4 (8.2)	9	1 (2.1)	1
Urethral pain	7 (14.3)	22	2 (4.3)	5	6 (12.2)	21	2 (4.3)	5
AEs at the time of administration								
Total	30 (61.2)	100	15 (31.9)	26	29 (59.2)	99	13 (27.7)	23
Postprocedural hematuria	0 (0.0)	0	1 (2.1)	1	0 (0.0)	0	0 (0.0)	0
Groin pain	1 (2.0)	1	0 (0.0)	0	1 (2.0)	1	0 (0.0)	0
Bladder discomfort	4 (8.2)	6	1 (2.1)	1	4 (8.2)	6	1 (2.1)	1
Bladder irritation	5 (10.2)	8	1 (2.1)	1	5 (10.2)	8	1 (2.1)	1
Bladder pain	15 (30.6)	47	10 (21.3)	15	15 (30.6)	47	9 (19.1)	13
Dysuria	2 (4.1)	3	0 (0.0)	0	2 (4.1)	3	0 (0.0)	0
Hematuria	0 (0.0)	0	2 (4.3)	2	0 (0.0)	0	2 (4.3)	2
Pollakiuria	4 (8.2)	9	1 (2.1)	1	4 (8.2)	9	1 (2.1)	1
Urethral pain	7 (14.3)	22	2 (4.3)	5	6 (12.2)	21	2 (4.3)	5
Bladder dysfunction	1 (2.0)	4	0 (0.0)	0	1 (2.0)	4	0 (0.0)	0
Severity of AEs								
Mild	33 (67.3)	115	22 (46.8)	43	26 (53.1)	86	12 (25.5)	22
Moderate	9 (18.4)	25	11 (23.4)	17	8 (16.3)	19	2 (4.3)	3
Severe	2 (4.1)	2	1 (2.1)	1	0 (0.0)	0	0 (0.0)	0

Analysis set: safety analysis set.

## Discussion

This randomized, placebo‐controlled, double‐blind parallel trial demonstrated that, compared to placebo, intravesical instillation of KRP‐116D in bladder‐centric IC/BPS patients improved PROs, including IC/BPS symptoms and bladder pain, objective outcomes, including voiding parameters, and GRA scores. The treatment was also well tolerated.

Several studies have reported that intravesical DMSO was effective in relieving IC/BPS symptoms.^14^, ^17^ Among them, only one randomized placebo‐controlled crossover study in 33 IC/BPS patients demonstrated that DMSO had a significant clinical benefit over placebo.^18^ However, the UK National Institute for Health and Care Excellence concluded that the evidence level in this crossover study was inadequate due to the lack of information on the methods employed for randomization, blinding and statistical analyses.^19^ The Cochrane review also reported^20^ that the evidence base for treating IC/BPS with intravesical DMSO is limited, and randomized controlled trials are still needed. Our trial is, therefore, the first well‐controlled trial to demonstrate the clinical benefits of intravesical instillation of 50% DMSO in IC/BPS patients with the bladder‐centric phenotype.

There is still no global consensus on the terminology or diagnostic criteria for IC/BPS.^11^ The Japanese Guideline recommends cystoscopy for the initial diagnosis of IC/BPS with a differential diagnosis of either Hunner or non‐Hunner type IC/BPS.^1^ By contrast, in the International Society for the Study of Bladder Pain Syndrome Guideline^3^ and the American Urological Association Guideline,^2^ IC/BPS is diagnosed on the basis of symptoms, and cystoscopy is not recommended in uncomplicated cases. In 2017, the U.S. Food and Drug Administration Bone, Reproductive, and Urologic Drugs Advisory Committee updated the definition and criteria necessary for enrolment and outcomes required for clinical trials evaluating interventions for IC/BPS. The committee members unanimously agreed that patients with bladder‐centric IC and extra‐bladder BPS can be combined in one study protocol,^21^ while the importance of cystoscopy for establishing definitions and the diagnosis for IC/BPS was not discussed. However, in 2018, the International Consultation Interstitial Cystitis Japan meeting concluded that Hunner lesion IC/BPS with significant inflammation in the bladder is clinically and pathologically distinct from non‐Hunner IC/BPS, and can be categorized as a separate disease from non‐Hunner IC/BPS.^10^, ^11^, ^22^ It has also been reported that anti‐inflammatory action is one of the major mechanisms inducing therapeutic efficacy of DMSO in IC/BPS patients.^14^ Thus, the high incidence of Hunner lesions in our study population (Table 1) may have contributed to the clinical efficacy of DMSO shown in the present study although further studies are needed to clarify this point.

In the present study, the superiority of KRP‐116D over placebo, in terms of both objective and subjective outcomes, was confirmed. PRO data are regarded as significant information for real‐world practice for the treatment of IC/BPS as well as overactive bladder syndrome. Also, in recent clinical trials, GRA has been used as a primary endpoint; however, previous trials of IC/BPS or overactive bladder using GRA as a primary endpoint failed to meet the criteria for IC/BPS.^23^, ^24^ In the present study, it is noted that effectiveness of KRP‐116D was confirmed not only with regard to PROs such as IC symptoms and problem questionnaires and bladder pain score, but also with regard to GRA. Because GRA is a measure of patient’s satisfaction with treatment outcome, the superior efficacy of KRP‐116D over placebo in the GRA indicates that the treatment effect of KRP‐116D is clinically meaningful. Furthermore, our findings suggest that cystoscopic identification of bladder pathology such as Hunner lesions and confirmation of short‐term bladder pain relief with intravesical lidocaine are useful in recruiting patients with the bladder‐centric IC/BPS phenotype for clinical trials. In addition, a recent review article suggested that bladder glomerulations with hydrodistention‐induced mucosal bleeding are not a specific finding of IC/BPS.^22^ Therefore, confirmation of bladder‐derived pain with intravesical lidocaine seems to be particularly useful for enrolling patients with non‐Hunner bladder pathologies such as hydrodistention‐induced glomerulations. Furthermore, because bladder instillation of lidocaine solution was performed in both placebo and KRP‐116D groups and its pain‐reducing effects were short‐lasting, the superiority of KRP‐116D over placebo in both PROs and objective voiding parameters is likely to indicate that the therapeutic effects were induced by DMSO, but not by lidocaine.

Regarding safety, the incidence of ADRs in the KRP‐116D group was higher than in the placebo group (KRP‐116D; 59.2% *vs* placebo; 27.7%), but most ADRs were the result of bladder stimulation after the instillation treatment. The incidence decreased to a level similar to that of placebo after repeated administrations. Ocular toxicities have been reported as a consequence of both oral and dermal treatments with DMSO.^25^ However, no clinically significant changes were found in our study. The package insert of Rimso‐50^®^ states that the patient may notice a garlic‐like taste within a few minutes following instillation and this taste can persist for several hours, and breath and skin odour may remain for up to 72 h.^26^ In a previous clinical trial of intravesical KRP‐116D in healthy Japanese patients, garlic‐like breath odour was the only ADR, with an incidence of 66.7%.^27^ In contrast, the incidence of odour‐related ADRs in the present study was very low, at 4.1%. In the present study, these ADRs were only observed on the first day of administration and were not apparent by the time the following visit took place. It should be noted that the events were observed as objective findings by the investigators and/or study staff, but were not noticed by the patients themselves in either study. All healthy participants in the previous KRP‐116D study remained hospitalized throughout the study period.^27^ In contrast, the participants in this phase 3 study were outpatients, suggesting that the incidence depends heavily on the situation, such as presence/absence of study staff and study environment, under which patients are observed.

This study has some limitations. First, a weakness in the blinding was predicted due to the characteristic garlic‐like breath odour in patients receiving DMSO.^14^, ^28^ However, the incidence of garlic‐like breath was very low (4.1%) in this study. Blinding would not seem to have been compromised by KRP‐116D‐derived odour; thus, we believe that the blind trial characteristics were maintained. Second, the number of patients might have been too small to fully evaluate safety. However, intravesical 50 mL of 50% DMSO has been used for over 40 years in IC/BPS patients worldwide on an off‐label basis in real‐world practice,^1^, ^29^ suggesting that physicians are familiar with the safety profile. Finally, the short duration of this trial (12 weeks) may be a limitation. Studies to investigate the long‐term efficacy and safety of KRP‐116D are needed.

In conclusion, our trial is the first well‐controlled clinical study to demonstrate that, compared to placebo, intravesical instillation of KRP‐116D in IC/BPS patients improved both PROs and objective voiding parameters. KRP‐116D was well tolerated, and most TEAEs were mild to moderate, and manageable. Furthermore, careful phenotyping and selection of patients suited to the clinical trial objectives seems particularly important for the success of clinical trials in IC/BPS patients due to their heterogeneous pathological backgrounds.

## Conflict of interest

N. Yoshimura and T. Ueda report personal fees from Kyorin, during the conduct of the study. N. Yoshimura, Y. Homma, H. Tomoe, A. Otsuka, T. Kitta, A. Niimi, M. Takei and K. Ameda report consulting fees for designing the study. All the authors except N. Yoshimura, Y. Homma, T. Uno and A. Ohinata report clinical investigation expenses for conducting the study. With regard to outside the submitted work, N. Yoshimura reports grants from Astellas, and personal fees from Kyorin, Astellas and Nippon Shinyaku. Y. Homma reports personal fees from Kyorin, Astellas, Kissei, Pfizer Japan, Nippon Shinyaku, Takeda and Daiichi Sankyo. H. Tomoe reports grants from Astellas, Pfizer Japan, and personal fees from Kyorin, Astellas, Kissei, Pfizer Japan, Nippon Shinyaku, Ono, Takeda and Hisamitsu. A. Otsuka reports personal fees from Kyorin, Astellas, Kissei, Pfizer Japan, Nippon Shinyaku, Novartis and Sanofi. T. Kitta reports grants from Otsuka and personal fees from Kyorin, Astellas, Kissei, Pfizer Japan, Nippon Shinyaku, Asahikasei, Otsuka and Ono. N. Masumori reports grants from Astellas, Ono, Takeda, Daiichi Sankyo and Kissei, and personal fees from Astellas, Kissei, Takeda, Janssen and Astra Zeneca. T. Mitsui reports grants from Kyorin and Daiichi Sankyo, and personal fees from Kyorin, Astellas, Kissei, Pfizer Japan, Nippon Shinyaku and Astra Zeneca. M. Nanri reports personal fees from Kyorin. M. Takei reports personal fees from Astellas, Kissei, Pfizer Japan, Nippon Shinyaku and Taiho. S. Kobayashi reports personal fees from Kissei. S. Kageyama reports personal fees from Kyorin, Astellas, Kissei, Pfizer Japan, Kobayashi and Glaxo. K. Kokura reports personal fees from Astellas, Pfizer Japan and Nippon Shinyaku. T. Uno and A. Ohinata are employees of Kyorin Pharmaceutical Co., Ltd. T. Ueda reports personal fees from Astellas, Kissei, Nippon Shinyaku, Ono, Bayer, Taiho, Daiichi Sankyo, Hisamitsu, Nippon Chemiphar and Seikagaku. Y. Akiyama, A. Niimi, T. Namima, M. Takei, A. Ymaguchi, Y. Sekiguchi, M. Kajiwara, M. Ameda, Y. Ohashi, O. Muraki, H. Okazoe, T. Yamanishi and T. Watanabe report no competing interests.

## Supporting information


**Figure S1**. Study design.
**Table S1**. Inclusion and exclusion criteria.
**Table S2**. Efficacy variables at weeks 0, 4, 8, and 12, and change in efficacy variables from baseline (week 0).
**Table S3**. Time to onset and resolution of AEs at the time of administration.
