# Supplementary material for: Efficacy and safety of intravesical instillation of KRP‐116D (50% dimethyl sulfoxide solution) for interstitial cystitis/bladder pain syndrome in Japanese patients: A multicenter, randomized, double‐blind, placebo‐controlled, clinical study
Source: Int J Urol. 2021 Feb 12;28(5):545–53. doi: 10.1111/iju.14505 (PMC8247858; doi:10.1111/iju.14505)
Supplement: Supplementary file 1 — Figure S1. Study design. Table S1. Inclusion and exclusion criteria. Table S2. Efficacy variables at weeks 0, 4, 8, and 12, and change in efficacy variables from baseline (week 0). Table S3. Time to onset and resolution of AEs at the time of administration. [file IJU-28-545-s001.docx]

**Supplementary Materials**

**Supplementary Figure S1** – Study design

**Supplementary Table S1** – Inclusion and exclusion criteria

**Supplementary Table S2** – Efficacy variables at week 0, 4, 8, and 12, and change in efficacy variables from baseline (week 0)

**Supplementary Table S3** – Time to onset and resolution of adverse events at the time of administration

**Supplementary Figure S1 – Study design**


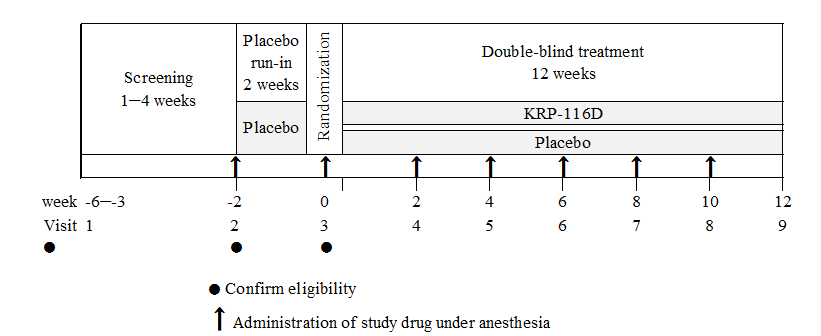


**Supplementary Table S1 – Inclusion and exclusion criteria**

| **Inclusion criteria** | **Exclusion criteria** |
| --- | --- |
| ***Screening (Visit 1)*** | |
| - Japanese outpatients of 20 years of age or older at the time of informed consent - Currently diagnosed with interstitial cystitis by cystoscopy - Had bladder pain and other interstitial cystitis symptoms (e.g. urinary frequency, persistent urge to urinate and/or urinary urgency) - Able to follow the investigator’s dietary advice during the study period (New dietary guidance will begin at Visit 1 if it has not been implemented prior to Visit 1) - Understands the purpose of this study as explained by the investigator, and that their participation is voluntary and they are free to withhold consent or withdraw from the study at any time, and the investigators determined that the patient is suitable for participation in the study | **General**   - Determined by the investigators as not suitable for participation in the study - Currently diagnosed with cancer, or have previous history of cancer within the preceding 5 years - Currently diagnosed with severe heart, liver, kidney, or blood disorder - Have previous history of allergy to dimethyl sulfoxide (DMSO) and/or amide type anaesthetics - Participated in any other clinical study within 12 week prior to Visit 1, or currently participating in a clinical trial - Currently diagnosed with posterior subcapsular cataract and/or atopic cataract - Lens surgery such as intraocular lens implantation is planned during study period - Patients who are pregnant, pregnant women or lactating women or women who desire to become pregnant and those who will not agree to use a highly effective method of contraception during the study period   **Urology related**   - Have previous history of urinary infection (e.g. bacterial cystitis, bladder tuberculosis, urethritis, prostatitis, genital chlamydia infection, and genital herpes) within 12 week prior to Visit 1 - Currently diagnosed with any of following diseases, and/or current urinary symptoms (i.e. bladder pain, bladder discomfort, urinary frequency, persistent urge to urinate, and/or urinary urgency) are caused primarily by these diseases:   - Bladder diseases (overactive bladder, neurogenic bladder, bladder stone, radiation cystitis)   - Prostate/urethral diseases (benign prostatic hyperplasia, urethral diverticulum, urethral stricture, urethral stone)   - Gynaecological diseases (endometriosis, uterine fibroids, vaginitis, menopausal syndrome, pelvic organ prolapse)   - Others (neurogenic urinary frequency, polyuria) - Have previous history of augmentation cystoplasty or cystectomy - Have previous history of chemical compound (such as cyclophosphamide) derived cystitis   **Therapy and drug related**   - Have history of the following therapies within 24 week prior to Visit 1:   Hydrodistention, intravesical laser therapy, intravesical electrical coagulation, transurethral resection, pelvic reconstructive surgery, nerve block or spinal cord stimulation for pain relief   - Received intravesical DMSO instillation within 24 week prior to Visit 1 - Received intravesical instillation other than DMSO within 12 week prior to Visit 1 - Have previously participated in this study, and have been treated with investigational drug |
| ***Placebo run-in (Visit 2)*** | |
| - Able to keep symptom dairy for 2 consecutive days (3 days for bladder pain) before Visit 2 - Have a micturition volume of 50 mL or more during the symptom diary period - Have 24-h micturition volume of less than 3000 mL during the symptom diary period - Have an average score of 4 points or more on the numerical rating scale (NRS) for bladder pain confirmed during the symptom diary period - Have a score of 9 points or more on the O’Leary-Sant interstitial cystitis symptom index (ICSI) - Have a residual urine volume of less than 100 mL - Able to adhere to the study restrictions regarding prohibited therapies/medications, concomitant medications - Had no symptoms suggestive of urinary tract infections (such as bacterial cystitis, bladder tuberculosis, urethritis, prostatitis, genital chlamydia infection, and genital herpes) during the screening period - No clinically problematic abnormalities revealed in the ophthalmic test - Not diagnosed with posterior subcapsular cataract or atopic cataract by ophthalmic test - Determined by the investigators to be able to continue in this study |  |
| ***Randomization (Visit 3)*** | |
| - Able to keep a symptom dairy for 2 consecutive days (3 days for bladder pain) before Visit 3 - Have a 24-h urinary frequency of 8 times or more during the symptom diary period - Both of the following items are met for the ICSI:   (1) ≥9 points  (2) No decrease in the ICSI by ≥30% from that at Visit 2   - Have an average score of 4 points or more on NRS for bladder pain confirmed during the symptom diary period - Able to adhere to the study restrictions regarding prohibited therapies/medications, concomitant medications - Had no symptoms suggestive of urinary tract infections (such as bacterial cystitis, bladder tuberculosis, urethritis, prostatitis, genital chlamydia infection, and genital herpes) before Visit 3 - Pain has been confirmed to originate in the bladder by lidocaine instillation treatment given at Visit 2 by the investigators. (Whether lidocaine treatment administered at Visit 2 on bladder pain was effective was assessed during Visit 2.) - Determined by the investigators to be able to safely continue in this study |  |

DMSO, dimethyl sulfoxide; ICSI, O’Leary-Sant interstitial cystitis symptom index; NRS, numerical rating scale

**Supplementary Table S2 – Efficacy variables at weeks 0, 4, 8, and 12, and change in efficacy variables from baseline (week 0)**

|  |  | Week 0 | | | Week 4 | | | Week 8 | | | Week 12 | | |
| --- | --- | --- | --- | --- | --- | --- | --- | --- | --- | --- | --- | --- | --- |
|  |  | n | Mean | SD | n | Mean | SD | n | Mean | SD | n | Mean | SD |
| ICSI total score | KRP-116D | 49 | 13.9 | 3.1 | 48 | 11.1 | 4.1 | 48 | 9.5 | 3.9 | 48 | 8.7 | 4.2 |
|  | Placebo | 47 | 13.7 | 3.1 | 47 | 11.6 | 3.4 | 46 | 11.2 | 3.7 | 45 | 10.3 | 4.0 |
|  | Estimated LS mean difference between the groups (95% CI) | ‒ | | | -0.7 (-1.9, 0.5) | | | -1.9 (-3.0, -0.7) | | | -1.8 (-3.3, -0.3) | | |
|  | *p*-value |  | | | 0.2493 | | | 0.0022 | | | 0.0188 | | |
| ICPI total score | KRP-116D | 49 | 12.0 | 3.2 | 48 | 9.6 | 4.2 | 48 | 8.0 | 4.1 | 48 | 7.1 | 4.6 |
|  | Placebo | 47 | 11.8 | 3.0 | 47 | 10.6 | 3.6 | 46 | 10.2 | 3.5 | 45 | 9.5 | 3.9 |
|  | Estimated LS mean difference between the groups (95% CI) | ‒ | | | -1.2 (-2.2, -0.1) | | | -2.3 (-3.5, -1.2) | | | -2.5 (-4.0, -1.0) | | |
|  | *p*-value |  | | | 0.0266 | | | 0.0002 | | | 0.0014 | | |
| Number of micturitions/24 h | KRP-116D | 49 | 16.41 | 9.41 | 48 | 14.17 | 6.95 | 48 | 12.76 | 5.11 | 48 | 12.35 | 4.92 |
|  | Placebo | 47 | 14.93 | 5.72 | 47 | 13.79 | 5.28 | 46 | 13.64 | 5.40 | 45 | 13.59 | 6.18 |
|  | Estimated LS mean difference between the groups (95% CI) | ‒ | | | -0.57 (-1.89, 0.75) | | | -1.79 (-3.04, -0.54) | | | -2.11 (-3.62, -0.60) | | |
|  | *p*-value |  | | | 0.3930 | | | 0.0054 | | | 0.0068 | | |
| Voided volume per micturition (mL) | KRP-116D | 49 | 109.4 | 54.8 | 48 | 130.9 | 67.9 | 48 | 139.4 | 74.6 | 48 | 148.0 | 81.8 |
|  | Placebo | 47 | 114.1 | 58.5 | 47 | 118.3 | 48.8 | 46 | 127.1 | 59.2 | 45 | 128.0 | 59.5 |
|  | Estimated LS mean difference between the groups (95% CI) | ‒ | | | 17.4 (6.6, 28.1) | | | 17.1 (3.2, 31.0) | | | 24.8 (8.0, 41.6) | | |
|  | *p*-value |  | | | 0.0019 | | | 0.0166 | | | 0.0042 | | |
| Maximum voided volume per micturition (mL) | KRP-116D | 49 | 183.5 | 104.2 | 48 | 207.0 | 100.7 | 48 | 224.7 | 111.9 | 48 | 226.8 | 114.7 |
|  | Placebo | 47 | 184.4 | 104.0 | 47 | 181.9 | 84.0 | 46 | 203.6 | 111.9 | 45 | 194.1 | 97.0 |
|  | Estimated LS mean difference between the groups (95% CI) | ‒ | | | 26.3 (11.0, 41.6) | | | 22.3 (3.7, 41.0) | | | 34.8 (12.8, 56.8) | | |
|  | *p*-value |  | | | 0.0010 | | | 0.0196 | | | 0.0023 | | |
| Change in maximum voided volume per micturition (%) | KRP-116D | --- | --- | --- | 48 | 206.98 | 100.68 | 48 | 224.69 | 111.93 | 48 | 226.82 | 114.71 |
|  | Placebo | --- | --- | --- | 47 | 181.91 | 83.99 | 46 | 203.64 | 111.91 | 45 | 194.06 | 97.02 |
|  | Estimated LS mean difference between the groups (95% CI) | ‒ | | | 13.77 (3.41, 24.13) | | | 15.65 (4.54, 26.76) | | | 18.35 (4.34, 32.35) | | |
|  | *p*-value |  | | | 0.0098 | | | 0.0063 | | | 0.0108 | | |
| Numerical rating scale for pain | KRP-116D | 49 | 6.50 | 1.46 | 48 | 4.54 | 2.13 | 48 | 3.94 | 2.29 | 48 | 3.64 | 2.49 |
|  | Placebo | 47 | 6.51 | 1.50 | 47 | 5.40 | 2.02 | 46 | 5.01 | 2.00 | 45 | 4.45 | 2.28 |
|  | Estimated LS mean difference between the groups (95% CI) | ‒ | | | -0.86 (-1.62, -0.10) | | | -1.03 (-1.81, -0.24) | | | -0.78 (-1.71, 0.15) | | |
|  | *p*-value |  | | | 0.0261 | | | 0.0112 | | | 0.0973 | | |
| Analysis set: Full analysis set (FAS)  CI=confidence interval; ICSI=O’Leary-Sant interstitial cystitis symptom index; ICPI=O’Leary-Sant interstitial cystitis problem index; LS mean=least-squares mean; SD=standard deviation | | | | | | | | | | | | | |

**Supplementary Table S3 –Time to onset and resolution of adverse events at the time of administration**

|  |  | Treatment-emergent adverse events | |  | Adverse drug reactions | |
| --- | --- | --- | --- | --- | --- | --- |
|  |  | KRP-116D | Placebo |  | KRP-116D | Placebo |
| Total | Number of subjects | 49 | 47 |  | 49 | 47 |
|  | Number (%) with TEAE | 30 (61.2) | 15 (31.9) |  | 29 (59.2) | 13 (27.7) |
|  | Number of events | 100 | 26 |  | 99 | 23 |
|  | Time to onset (day) | 1 (1 - 2) | 1 (1 - 13) |  | 1 (1 - 2) | 1 (1 - 2) |
|  | Time to resolution (day) | 2 (1 - 50) | 1 (1 - 27) |  | 2 (1 - 50) | 1 (1 - 27) |
| Week 0 - Week 2 | Number of subjects | 49 | 47 |  | 49 | 47 |
|  | Number (%) with TEAE | 26 (53.1) | 11 (23.4) |  | 25 (51.0) | 9 (19.1) |
|  | Number of events | 29 | 13 |  | 28 | 11 |
|  | Time to onset (day) | 1 (1 - 2) | 1 (1 - 6) |  | 1 (1 - 2) | 1 (1 - 2) |
|  | Time to resolution (day) | 2 (1 - 12) | 3 (1 - 27) |  | 2 (1 - 12) | 3 (1 - 27) |
| Week 2 - Week 4 | Number of subjects | 49 | 47 |  | 49 | 47 |
|  | Number (%) with TEAE | 15 (30.6) | 3 (6.4) |  | 15 (30.6) | 3 (6.4) |
|  | Number of events | 19 | 3 |  | 19 | 3 |
|  | Time to onset (day) | 1 (1 - 1) | 1 (1 - 1) |  | 1 (1 - 1) | 1 (1 - 1) |
|  | Time to resolution (day) | 2 (1 - 3) | 1 (1 - 2) |  | 2 (1 - 3) | 1 (1 - 2) |
| Week 4 - Week 6 | Number of subjects | 49 | 47 |  | 49 | 47 |
|  | Number (%) with TEAE | 13 (26.5) | 3 (6.4) |  | 13 (26.5) | 3 (6.4) |
|  | Number of events | 17 | 3 |  | 17 | 3 |
|  | Time to onset (day) | 1 (1 - 2) | 1 (1 - 1) |  | 1 (1 - 2) | 1 (1 - 1) |
|  | Time to resolution (day) | 1 (1 - 50) | 1 (1 - 1) |  | 1 (1 - 50) | 1 (1 - 1) |
| Week 6 - Week 8 | Number of subjects | 49 | 47 |  | 49 | 47 |
|  | Number (%) with TEAE | 12 (24.5) | 3 (6.4) |  | 12 (24.5) | 2 (4.3) |
|  | Number of events | 15 | 3 |  | 15 | 2 |
|  | Time to onset (day) | 1 (1 - 1) | 1 (1 - 13) |  | 1 (1 - 1) | 1 (1 - 1) |
|  | Time to resolution (day) | 1 (1 - 8) | 1 (1 - 1) |  | 1 (1 - 8) | 1 (1 - 1) |
| Week 8 - Week 10 | Number of subjects | 49 | 47 |  | 49 | 47 |
|  | Number (%) with TEAE | 11 (22.4) | 2 (4.3) |  | 11 (22.4) | 2 (4.3) |
|  | Number of events | 13 | 2 |  | 13 | 2 |
|  | Time to onset (day) | 1 (1 - 1) | 1 (1 - 1) |  | 1 (1 - 1) | 1 (1 - 1) |
|  | Time to resolution (day) | 1 (1 - 12) | 1 (1 - 1) |  | 1 (1 - 12) | 1 (1 - 1) |
| Week 10 - Week 12 | Number of subjects | 49 | 46 |  | 49 | 46 |
|  | Number (%) with TEAE | 6 (12.2) | 2 (4.3) |  | 6 (12.2) | 2 (4.3) |
|  | Number of events | 7 | 2 |  | 7 | 2 |
|  | Time to onset (day) | 1 (1 - 1) | 1 (1 - 1) |  | 1 (1 - 1) | 1 (1 - 1) |
|  | Time to resolution (day) | 1 (1 - 2) | 2 (1 - 3) |  | 1 (1 - 2) | 2 (1 - 3) |
| Analysis set: Safety analysis set (SAF)  Time to onset of the adverse event was defined as the number of days from the last administration of the study drug. Median (minimum – maximum)  Time (days) to resolution was defined as the number of days from the onset day of the adverse event to the day of resolution. Median (minimum – maximum)  TEAE=Treatment-emergent adverse event | | | | | | |
